# Methods for selecting the best evidence to inform a NICE technology appraisal on selective internal radiation therapies for hepatocellular carcinoma

**DOI:** 10.1186/s13643-020-01447-x

**Published:** 2020-08-16

**Authors:** Ros Wade, Sahar Sharif-Hurst, Melissa Harden, Matthew Walton, Lindsay Claxton, Robert Hodgson, Alison Eastwood

**Affiliations:** Centre for Reviews and Dissemination, University of York, York, YO10 5DD UK

**Keywords:** Technology appraisal, Systematic review, Study selection, Critical appraisal, Selective internal radiation therapy, Hepatocellular carcinoma

## Abstract

**Background:**

Systematic reviews of medical devices are particularly challenging as the quality of evidence tends to be more limited than evidence on pharmaceutical products. This article describes the methods used to identify, select and critically appraise the best available evidence on selective internal radiation therapy devices for treating hepatocellular carcinoma, to inform a technology appraisal for the National Institute for Health and Care Excellence.

**Methods:**

A comprehensive search of ten medical databases and six grey literature sources was undertaken to identify studies of three devices (TheraSphere®, SIR-Spheres® and QuiremSpheres®) for treating hepatocellular carcinoma. The large evidence base was scoped before deciding what level of evidence to include for data extraction and critical appraisal. The methodological quality of the included studies was assessed using criteria relevant to each study design.

**Results:**

Electronic searches identified 4755 records; over 1000 met eligibility criteria after screening titles and abstracts. A hierarchical process was used to scope these records, prioritising comparative studies over non-comparative studies, where available. One hundred ninety-four full papers were ordered; 64 met the eligibility criteria. For each intervention, studies were prioritised by study design and applicability to current UK practice, resulting in 20 studies subjected to critical appraisal and data extraction. Only two trials had a low overall risk of bias. In view of the poor quality of the research evidence, our technology appraisal focused on the two higher quality trials, including a thorough critique of their reliability and generalisability to current UK practice. The 18 poorer quality studies were briefly summarised; many were very small and results were often contradictory. No definitive conclusions could be drawn from the poorer quality research evidence available.

**Conclusions:**

A systematic, pragmatic process was used to select and critically appraise the vast quantity of research evidence available in order to present the most reliable evidence on which to develop recommendations.

**Systematic review registration:**

PROSPERO CRD42019128383.

## Background

The National Institute for Health and Care Excellence (NICE) uses the best available evidence to develop recommendations that guide decisions in health, public health and social care. Technology appraisals assess the clinical and cost-effectiveness of health technologies, including pharmaceutical products, procedures and devices, to develop recommendations on their use within the NHS. This ensures that all patients have equitable access to the most clinically and cost-effective treatments available. Single technology appraisals assess a single drug or treatment, whereas multiple technology appraisals assess several drugs or treatments used for the same condition [1].

The process of undertaking a NICE technology appraisal involves identifying relevant evidence to inform UK practice, relating to a specific scope issued by NICE. Systematic searches aim to identify all relevant evidence to inform indirect comparisons and economic analyses to estimate the relative clinical and cost-effectiveness of the technologies being appraised. The highest quality evidence contributes directly to the formal assessment of clinical and cost-effectiveness, whilst lower quality evidence is presented as supporting evidence within the appraisal, hence the requirement for prioritisation of the evidence identified.

This article describes the methods used to identify, select and critically appraise the best available clinical effectiveness evidence in a systematic review conducted for a multiple technology appraisal of selective internal radiation therapies (SIRT) for treating hepatocellular carcinoma (HCC). Recommendations on the use of SIRT within the NHS are being developed on the basis of this appraisal, which also included an economic model, informed by the clinical effectiveness review, to assess the cost-effectiveness of SIRT. The NHS is legally obliged to fund and resource medicines and treatments recommended by NICE’s technology appraisals.

HCC is the most common type of primary liver cancer. The majority of HCCs are associated with a known underlying aetiology; in the UK, the underlying aetiology is commonly alcohol-related liver disease or non-alcoholic fatty liver disease, whilst in non-Western populations, viral hepatitis infection is the primary cause of HCC [2]. Clinical management of HCC is complex, owing to the reduced liver function of patients resulting from both the underlying liver disease and the growing tumour. The Barcelona Clinic Liver Cancer (BCLC) staging system is used to establish prognosis and enable the selection of appropriate treatment based on the combination of tumour burden, liver function and performance status. Patients are classified into five stages: (0) very early stage, (A) early stage, (B) intermediate stage, (C) advanced stage and (D) terminal stage [3].

The primary aim of therapy in patients with very early or early stage HCC is typically curative, including surgery (resection or transplant) or ablation. Conventional transarterial therapies, which deliver an embolising agent (alone or in combination with chemotherapy) through the hepatic artery, are the standard of care for patients with intermediate stage disease, where resection or other curative treatment modalities are unsuitable. In patients with advanced HCC, or who have previously failed on conventional transarterial therapies, the current standard of care is systemic therapy (sorafenib, lenvatinib or regorafenib). Best supportive care is offered to patients when conventional transarterial therapies or systemic therapy is not available or appropriate, including patients with terminal stage disease.

SIRT deliver radiation directly to liver tumours via microspheres that are injected into the hepatic artery. There are currently three commercially available SIRT technologies: TheraSphere®, SIR-Spheres® and QuiremSpheres®. Each of the SIRT technologies are CE marked class III active medical devices but they differ in their composition. TheraSphere® are glass microspheres containing yttrium-90 radioactive isotopes, SIR-Spheres® are resin microspheres containing yttrium-90 radioactive isotopes and QuiremSpheres® are poly-l-lactic acid microspheres containing holmium-166 radioactive isotopes. Our clinical advisors considered that the most likely position for SIRT in the HCC treatment pathway is for patients with intermediate or advanced stage HCC as a non-curative treatment option.

In contrast to pharmaceutical product development, where medicines have to be rigorously evaluated for safety and efficacy (regulated by the European Medicines Agency in Europe and the Food and Drug Administration in the USA) before they can be placed on the market, medical devices undergo a conformity assessment to demonstrate that they are safe and perform as intended. Manufacturers can place a CE (Conformité Européenne) mark on a medical device once it has passed a conformity assessment. Evidence requirements for CE markings are limited and focussed primarily upon safety; medical devices are often evaluated using non-randomised studies or small single centre trials [4]. Therefore, selecting the best available clinical effectiveness evidence and critically appraising studies of medical devices can present particular challenges for systematic reviewers.

This article describes the systematic and pragmatic methods developed to select and critically appraise the large body of poor quality research evidence in a systematic review conducted for a NICE multiple technology appraisal of SIRT devices for treating HCC.

## Methods

### Protocol registration

The research protocol for the full multiple technology appraisal project was registered on PROSPERO, the international prospective register of systematic reviews in health and social care; registration number CRD42019128383.

### Search strategy

A comprehensive search was undertaken to systematically identify clinical effectiveness literature. The search strategy was developed in Ovid MEDLINE by an Information Specialist (MH) with input from the review team, including our clinical advisors (a hepatologist, an interventional radiologist and a clinical oncologist). The strategy consisted of a set of terms for HCC combined with terms for SIRT, limited to studies from the year 2000 onwards. The 2000 date limit was applied as scoping searches had identified controlled studies of SIR-Spheres® and TheraSphere® published after the year 2000; earlier studies were preliminary uncontrolled studies with limited value for assessing clinical effectiveness. In addition, clinical advice confirmed that treatment options available for patients prior to 2000 were different from those used in current UK practice. The searches were not limited by language or study design. The MEDLINE strategy was adapted for use in all other resources searched.

Ten electronic medical databases and six grey literature sources were searched on 28 January 2019 (MEDLINE, EMBASE, Cumulative Index to Nursing & Allied Health, Science Citation Index, Cochrane Central Register of Controlled Trials, Cochrane Database of Systematic Reviews, Database of Abstracts of Reviews of Effects, Health Technology Assessment database, NHS Economic Evaluations Database, EconLit, ClinicalTrials.gov, WHO International Clinical Trials Registry portal, EU Clinical Trials Register, PROSPERO, Conference Proceedings Citation Index – Science and ProQuest Dissertations & Theses A&I). The NICE website and NHS Evidence were searched for relevant guidelines. Evidence submissions from the manufacturers/sponsors of the three SIRT devices and relevant systematic reviews were also hand-searched to identify further relevant studies. Clinical advisors were consulted for any additional studies.

### Eligibility criteria

Studies of the selective internal radiation therapies TheraSphere®, SIR-Spheres® and QuiremSpheres® for patients with early, intermediate or advanced stage unresectable HCC were included in the review. The comparator was established clinical management without SIRT, conventional transarterial therapies, systemic therapy (sorafenib, lenvatinib and regorafenib) or best supportive care.

Randomised controlled trials (RCTs) were eligible for inclusion. However, where RCT evidence was lacking for a particular SIRT device, non-randomised comparative studies (including retrospective studies) and non-comparative studies were considered for inclusion. The evidence was scoped before deciding what level of evidence would be included for data extraction and critical appraisal.

### Study selection

Search results were imported into EndNote® software and de-duplicated. Studies were initially assessed for relevance by one reviewer examining titles and abstracts, with a second reviewer checking 10% of records. Full manuscripts of any titles/abstracts that appeared relevant were obtained where possible and the relevance of each study was assessed independently by two reviewers according to the eligibility criteria. Any discrepancies were resolved through consensus and, where necessary, a third reviewer was consulted. Relevant foreign language studies were translated and assessed for inclusion in the review. Studies available only as abstracts were included and attempts were made to contact authors for further data, where necessary.

### Critical appraisal

The methodological quality of the included studies was assessed using criteria relevant to the study design. RCTs were assessed using the most recent version of the Cochrane risk of bias tool [5]. Quality assessment tools for other study designs were developed using relevant criteria, such as those outlined in CRD’s guidance on undertaking systematic reviews [6]. Quality assessment tools were piloted on a small number of studies and refined prior to being used. Quality assessment was undertaken by one reviewer and independently checked by a second reviewer. Any disagreements were resolved through consensus and, where necessary, a third reviewer was consulted.

### Data synthesis

Characteristics of the included SIRT studies (such as participant and intervention characteristics, results and study quality) were tabulated. Pooling of trial data using meta-analytic techniques was planned, where sufficient clinically and statistically homogeneous data were available. Further details of the network meta-analyses and economic model, that were also presented to NICE as part of the multiple technology appraisal, are reported in an HTA report [7].

## Results

### Prioritisation for full paper screening

**Fig. 1:**
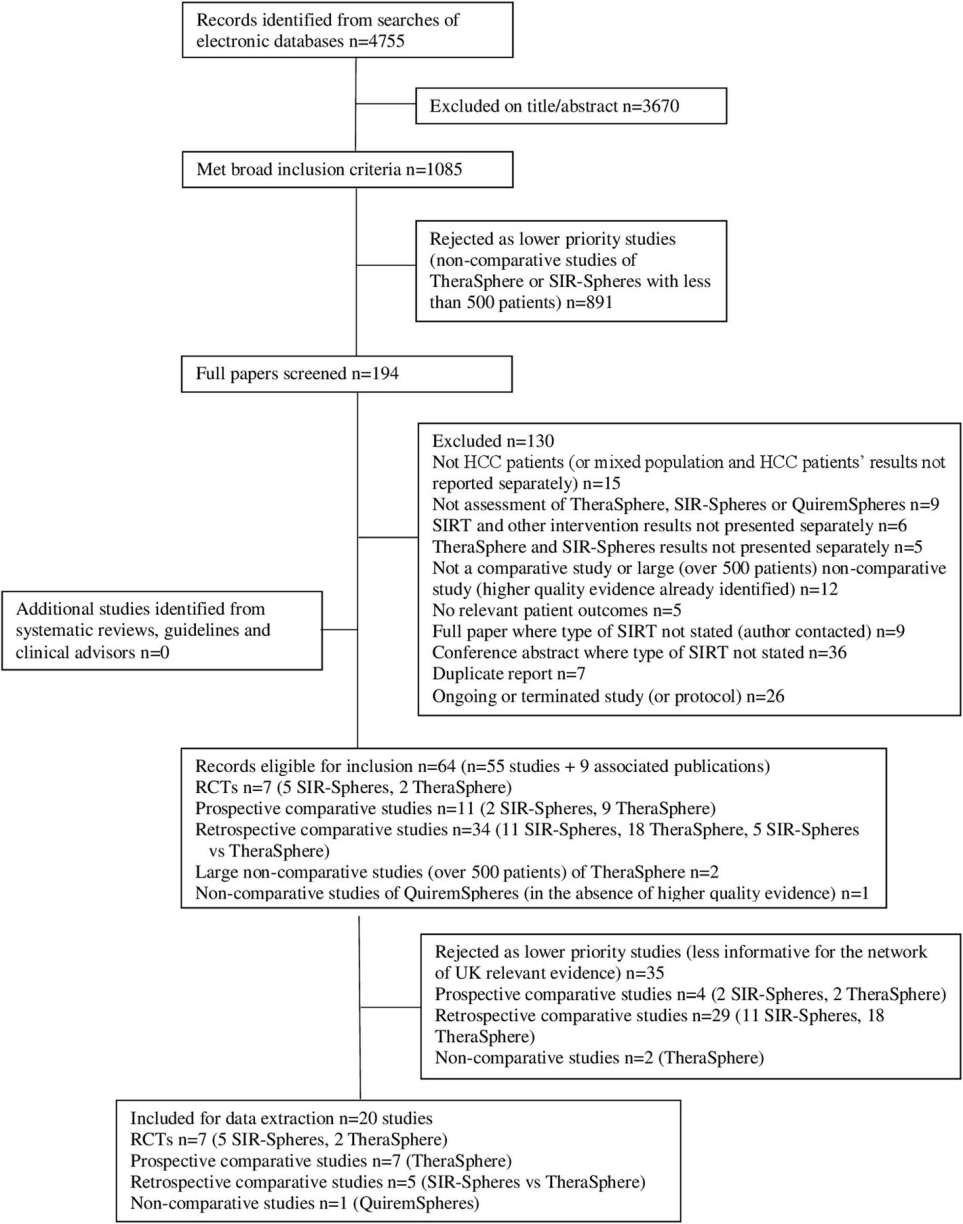
Flow diagram of the study selection process

The results of the study selection process are presented in a PRISMA diagram (Fig. 1). A total of 4755 records were identified by the electronic searches and imported into an EndNote® library, after de-duplication between databases. One reviewer (RW) screened 2615 titles and abstracts and another reviewer (SS) screened 2617 titles and abstracts; 477 records (10%) were double screened. Of the 4755 records, 3670 were excluded based on title and abstract screening. One thousand and eighty-five records appeared to meet the eligibility criteria for the review.

In view of the high number of potentially eligible records, the evidence was scoped in EndNote® before deciding which studies to order for full paper screening. Records were coded in terms of the intervention (type of SIRT and whether the study focussed on the delivery of SIRT or the work-up of patients prior to SIRT delivery), the study design (prospective or retrospective, comparative or non-comparative) and the number of HCC patients included in the study.

Comparative studies were prioritised over non-comparative studies, which have limited value in comparing the clinical and cost-effectiveness of different technologies. A total of 176 comparative studies appeared to meet the review inclusion criteria; therefore, all comparative studies were ordered for full paper screening: 47 RCTs, 26 prospective comparative studies and 103 retrospective comparative studies. However, it was clear that there were no comparative studies of the newer technology QuiremSpheres®; therefore, all studies that appeared to relate to QuiremSpheres® were ordered for full paper screening (*n* = 11). In addition, it was clear from scoping the Endnote® library that many of the comparative studies had small sample sizes (less than 100 patients). Therefore, large non-comparative studies that included over 500 patients were also ordered for full paper screening (*n* = 6). One final non-comparative study, in which BCLC subgroups and subsequent treatments were reported and which was considered to be particularly relevant for the economic model, was also ordered. Therefore, 194 records were ordered for full paper screening.

Of the 194 records ordered, 130 were excluded based on full paper screening and 64 records (55 studies plus 9 associated publications) were considered to be potentially relevant for inclusion in the clinical effectiveness review.

Hand searching the reference lists of evidence submissions from the manufacturers/sponsors of the three SIRT devices, relevant systematic reviews and guidelines identified by the electronic searches did not identify any additional relevant studies. Clinical advisors were not aware of any relevant studies not already identified by the electronic searches.

### Prioritisation for data extraction

In view of the high number of studies that met the eligibility criteria for the review and the limited time and resource available for reviewing studies, the most reliable and relevant studies were prioritised for data extraction and critical appraisal. For each intervention, studies were first prioritised by study design. There were five RCTs and two non-randomised prospective comparative studies of SIR-Spheres® compared with established clinical management without SIRT. There were two RCTs and nine non-randomised prospective comparative studies of TheraSphere® compared with established clinical management without SIRT. Therefore, 29 retrospective comparative studies of SIR-Spheres® or TheraSphere® and two non-comparative studies were not included for data extraction, since better quality data were available (see PRISMA diagram presented in Fig. 1). However, there were no prospective comparative studies directly comparing different SIRT technologies against each other; therefore, five retrospective comparative studies of SIR-Spheres® versus TheraSphere® were included. There were no comparative studies of QuiremSpheres®; therefore, one small non-comparative study was included, in the absence of any other research data.

After consultation with our clinical advisors, three prospective comparative studies that compared SIRT with therapies that are not applicable to current UK clinical practice were excluded: hepatic arterial chemotherapy (two studies) [8, 9] and 131 I-Lipiodol [10]. One further prospective comparative study was excluded because the dose of SIRT used in the study was not applicable to current UK practice [11].

Twenty of the 55 studies that met the review inclusion criteria were prioritised for data extraction: seven RCTs, seven prospective comparative studies, five retrospective comparative studies and one non-comparative case series. Basic study characteristics and results are summarised in Additional file 1. Because of the variable quality of the research evidence available and the differences between studies, in terms of patient and intervention characteristics and outcomes assessed, pairwise meta-analysis was not undertaken. A narrative synthesis of the quantity and quality of evidence on SIRT technologies was undertaken. Detailed results of the systematic review, network meta-analysis and economic model are reported in an HTA report [7].

### Risk of bias

Full risk of bias assessment results are presented in Additional file 2.

Two of the seven included RCTs had a low overall risk of bias [12, 13], assessed using the most recent version of the Cochrane risk of bias tool [5]. All other RCTs had either a high risk of bias [14–16] or some concerns regarding bias [17, 18]. There were concerns related to the randomisation process for each of the five lower quality RCTs, other concerns related to potential deviations from the intended interventions, measurement of the outcome and missing outcome data. All five lower quality RCTs had very small sample sizes (median 28, range 20 to 45).

All seven prospective comparative studies had a high risk of bias, assessed using a quality assessment tool developed specifically for this review [11, 19–24]. In particular, allocation to treatment groups was either inadequately described or inappropriate, resulting in baseline differences in prognostic factors between treatment groups in most studies. Most of the prospective comparative studies had small sample sizes (median 67, range 45 to 765).

Four of the five retrospective comparative studies directly comparing SIR-Spheres® with TheraSphere® had a high risk of bias, with baseline differences in prognostic factors between treatment groups [25–28]. One retrospective comparative study had an unclear risk of bias; it was unclear whether treatment groups were similar at baseline (due to lack of reporting of patient characteristics for separate treatment groups) or whether missing outcome data were balanced across treatment groups [29]. It was unclear whether outcome assessors were blinded to treatment group in any of the retrospective studies. All of the retrospective comparative studies had small sample sizes (median 77, range 17 to 97).

The small (*n* = 9) non-comparative case series of QuiremSpheres® was considered to be at a high risk of bias; it was unclear whether the patients included in the study were representative of those who would be eligible for SIRT in clinical practice and outcome measures were not consistently assessed [30].

The majority of research evidence on SIRT devices for the treatment of HCC was poor quality. Therefore, our multiple technology appraisal report focused on the two RCTs with a low overall risk of bias, presenting a detailed description of the results, as well as a thorough critique of the reliability of the results and their generalisability to current UK practice. One of the RCTs was conducted in France, whilst the other was conducted in the Asia-Pacific region. This has implications for the generalisability of the results to the UK population. HCC in European patients is more likely to be caused by alcohol or hepatitis C, whereas in Asia, it is more likely to be caused by hepatitis B. The natural history of these diseases is different, as are the treatment options.

Our report also briefly summarised the results of the poorer quality studies that were prioritised for data extraction; many of the studies were very small and results were often contradictory. No definitive conclusions could be drawn from the poorer quality research evidence available [7]. The network meta-analysis and economic model also focussed on the highest quality evidence, with lower quality studies only considered in scenario analyses.

## Discussion

Good quality research evidence is imperative for decision makers, such as NICE, to be able to develop clear recommendations to guide decisions in health, public health and social care. Medical devices are often evaluated using lower quality research designs, thus reducing our confidence in the reliability of the findings of the research. Assessing the clinical effectiveness and safety of medical devices presents particular challenges for systematic reviewers.

This article describes the methods used to systematically identify, select and critically appraise the best available research evidence on the clinical effectiveness of three SIRT devices (TheraSphere®, SIR-Spheres®, and QuiremSpheres®) for treating patients with unresectable early, intermediate or advanced stage HCC.

There is a large body of evidence on the clinical effectiveness and safety of SIRT technologies for the treatment of HCC. However, the vast majority of evidence is very low quality; only seven RCTs were identified, of which only two had a low risk of bias. The only studies identified that directly compared the different SIRT devices with each other were small retrospective comparative studies, all of which had a high or unclear risk of bias. The absence of good quality research evidence means that decision makers must rely on less robust findings from poorer quality studies. Most studies identified were small non-comparative studies, which have limited value for comparing the clinical and cost-effectiveness of different technologies. The sheer volume of small non-comparative data available meant that it was not possible to review all of the data within the timeframe available for undertaking this multiple technology appraisal. The poor quality of the majority of studies meant that their inclusion in the appraisal would not have provided reliable evidence for decision makers. Selecting the best available studies for each SIRT device highlighted the extremely poor quality of the data available for QuiremSpheres® in comparison with TheraSphere® and SIR-Spheres®; therefore, no conclusions could be drawn on the comparative clinical effectiveness of QuiremSpheres®.

The methods described may be informative to other research groups undertaking systematic reviews in areas where there is a large amount of research of uncertain quality. For this appraisal of multiple technologies for the treatment of patients with unresectable HCC, comparative studies were prioritised over non-comparative studies in order to present NICE with the most reliable information upon which to develop recommendations. Studies were also prioritised in terms of their applicability to current UK clinical practice. The most reliable and representative studies were assessed for bias using criteria relevant to each study design. Our multiple technology appraisal report focussed on two large RCTs with a low overall risk of bias, describing the results, a thorough critique of the reliability of each of the trial’s results and their generalisability to current UK practice. Poorer quality studies were briefly summarised, many of which were very small and results were often contradictory. Our methods may be particularly helpful for researchers undertaking systematic reviews of devices, where the quality of evidence is often poorer. Evidence requirements for CE markings are more limited than the requirements for pharmaceutical product development, in which medicines have to be rigorously evaluated for safety and efficacy. In addition, this paper may also provide insight to manufacturers of technologies developing primary studies of clinical effectiveness and safety.

The key strengths of the review were the comprehensive searches of the literature and the systematic and pragmatic approach used for scoping the vast quantity of research evidence available, incorporating both methodological and clinical expertise. The review included a detailed mapping and critical appraisal of the available research evidence on SIRT devices to ensure that the most reliable evidence was presented.

A limitation of the review was the lack of resource to double screen the titles and abstracts of all 4755 records in the Endnote® library; only 10% of records were double screened. However, all full papers retrieved for assessment against inclusion/exclusion criteria were independently screened by two reviewers. Hand searching of reference lists of relevant systematic reviews and guidelines and evidence submitted by the manufacturers/sponsors of the three SIRT devices, along with consultation with clinical advisors, did not identify any additional studies. This provides reassurance that no relevant studies were missed by the search strategy and screening procedure.

## Conclusions

There is a large body of poor quality research evidence on the clinical effectiveness of SIRT devices for the treatment of HCC. In view of the large body of evidence available, the most reliable and relevant studies were prioritised for data extraction and critical appraisal. Our multiple technology appraisal report focussed on the RCTs with a low overall risk of bias, briefly summarising the results of poorer quality studies that were prioritised for data extraction. The process used to select and critically appraise the vast quantity of research evidence available was complex and time consuming, but allowed us to present NICE with the most reliable evidence on which to develop recommendations.

## Supplementary information

**Additional file 1.** Study characteristics and results of the 20 studies prioritised for data extraction. (DOCX) Additional file 1 is a table presenting study characteristics and results of the 20 studies prioritised for data extraction.

**Additional file 2.** Risk of bias assessment results for the 20 studies prioritised for data extraction. (DOCX) Additional file 2 is a table presenting the risk of bias assessment results for the 20 studies prioritised for data extraction.

## Data Availability

All data relevant to this methodological article are included in this published article and its supplementary information files. Full results of the systematic review, network meta-analysis and economic model are reported in an HTA report (in press).

## Abbreviations

BCLC: Barcelona Clinic Liver Cancer
CE: Conformité Européenne
HCC: Hepatocellular carcinoma
NICE: National Institute for Health and Care Excellence
RCT: Randomised controlled trial
SIRT: Selective internal radiation therapies
