# Supplementary material for: Methods for selecting the best evidence to inform a NICE technology appraisal on selective internal radiation therapies for hepatocellular carcinoma
Source: Syst Rev. 2020 Aug 16;9:184. doi: 10.1186/s13643-020-01447-x (PMC7429468; doi:10.1186/s13643-020-01447-x)
Supplement: Supplementary file 1 — Additional file 1. Study characteristics and results of the 20 studies prioritised for data extraction. (DOCX) Additional file 1 is a table presenting study characteristics and results of the 20 studies prioritised for data extraction. [file 13643_2020_1447_MOESM1_ESM.docx]

**Additional File 1: Study characteristics and results of the 20 studies prioritised for data extraction**

| **Study** | **Study design/**  **location** | **Population** | **Intervention** | **Comparator** | **Brief results** | **Risk of bias** |
| --- | --- | --- | --- | --- | --- | --- |
| Vilgrain, 2017[13]  SARAH | RCT  France | Adults with locally advanced HCC (BCLC C) or new HCC not eligible for surgical resection, transplant or thermal ablation after a previously cured HCC (cured by surgery or thermoablative therapy) or HCC with two unsuccessful rounds of TACE | SIR-Spheres  (n=237) | Sorafenib  (n=222) | There was no statistically significant difference in overall survival or progression-free survival between treatment groups. More patients had a complete or partial response with SIR-Spheres (19%) than sorafenib (12%), although only 190 SIR-Spheres patients and 198 sorafenib patients were evaluable. Health-related quality of life was significantly better with SIR-Spheres than sorafenib, although the proportion of patients who completed the EORTC QLQ-C30 questionnaire was low. Adverse events were reported by more patients on sorafenib (94%) than SIR-Spheres (77%). | Low |
| Chow, 2018[12]  SIRveNIB | RCT  Asia-Pacific region | Adults with locally advanced HCC (BCLC B or C) not amenable to curative treatment | SIR-Spheres  (n=182) | Sorafenib  (n=178) | There was no statistically significant difference in overall survival, progression-free survival or health-related quality of life between treatment groups. More patients had a complete or partial response with SIR-Spheres (16.5%) than sorafenib (1.7%). Adverse events were reported by more patients on sorafenib (84.6%) than SIR-Spheres (60.0%). | Low |
| Kolligs, 2015[14]  SIR-TACE | RCT  Germany and Spain | Adults with unresectable liver-only HCC (without portal vein occlusion) | SIR-Spheres  (n=13) | TACE  (n=15) | There was no significant difference in progression-free survival between treatment groups (overall survival was not reported). More patients had a complete or partial response with SIR-Spheres (30.8%) than TACE (13.3%). Adverse events were reported by more patients on SIR-Spheres (92.3%) than TACE (66.7%). | High |
| Pitton, 2015[18] | RCT  Germany | Adults with unresectable N0, M0 HCC (BCLC stage B) | SIR-Spheres  (n=12) | DEB-TACE  (n=12) | Median overall survival was longer with DEB-TACE (788 days) than SIR-Spheres (592 days). Median progression-free survival was longer with DEB-TACE (216 days) than SIR-Spheres (180 days). Response rates and adverse event rates were not reported. | Some concerns |
| Ricke, 2015[15]  SORAMIC | RCT  Germany | Adults with unresectable intermediate or advanced HCC (BCLC stage B or C), with preserved liver function (Child-Pugh ≤B7) and ECOG <2, who were poor candidates for TACE (including those failing TACE) | SIR-Spheres plus sorafenib  (n=20) | Sorafenib alone  (n=20) | Overall survival, progression-free survival and response rates were not reported. Adverse event rates were similar between treatment groups. | High |
| Salem, 2016[16]  PREMIERE | RCT  USA | Adults with BCLC stage A/B unablatable/unresectable HCC with no vascular invasion, Child-Pugh A/B | TheraSphere  (n=24) | TACE  (n=21) | There was no significant difference in overall survival between treatment groups. Median time to progression was longer with TheraSphere (>26 months) than TACE (6.8 months). Response rates were similar between treatment groups. Adverse event rates were not reported. | High |
| Kulik, 2014[17] | RCT  USA | Adults with Child-Pugh ≤B8 and potential candidates for orthotopic liver transplant | TheraSphere  (n=10) | TheraSphere plus sorafenib  (n=10) | Overall survival was similar between treatment groups. Progression-free survival and response rates were not reported. The most commonly reported adverse events were reported by more patients on TheraSphere than TheraSphere plus sorafenib. | Some concerns |
| Kirchner, 2019[21] | Prospective comparative study  Germany | Adults with unresectable HCC | TheraSphere  (n=21) | TACE (n=33)  DEB-TACE  (n=13) | Overall survival and progression-free survival were not reported. Response rates were similar between treatment groups. There was a non-statistically significant difference in global health status/quality of life between groups, favouring TheraSphere. There was a significantly higher increase in fatigue after treatment with TACE compared with TheraSphere. Adverse event rates were not reported. | High |
| El Fouly, 2015[19] | Prospective comparative study  Germany and Egypt | Adults with intermediate stage (BCLC B) unresectable HCC and good liver function (Child-Pugh B <7) | TheraSphere  (n=44) | TACE  (n=42) | Median overall survival was slightly longer with TACE (18 months) than TheraSphere (16.4 months). Median time to progression was longer with TheraSphere (13.3 months) than TACE (6.8 months). More patients had a complete/partial response with TheraSphere (7%/68%) than TACE (5%/45%). The most commonly reported adverse event (abdominal pain) was reported by more patients on TACE (83%) than TheraSphere (5%). | High |
| Salem, 2013[23] | Prospective comparative study  USA | Adults with treatment naïve HCC with ECOG 0-2 | TheraSphere  (n=29) | TACE  (n=27) | Overall survival, progression-free survival and response rates were not reported. Most FACT-Hep quality of life scales showed a reduction in score in the TACE group with stability or increase in score in the TheraSphere group at 4 weeks. Almost all subscales favoured TheraSphere over TACE. Adverse event rates were not reported. | High |
| Memon, 2013[22] | Prospective comparative study  USA | Adults with HCC that progressed after intra-arterial locoregional therapies (TACE and SIRT) | TheraSphere  (n=42) | TACE  (n=54) | Overall survival, response rates and adverse event rates were not reported. Median time to progression was longer in the TheraSphere group (13.3 months) than the TACE group (8.4 months). | High |
| Hickey, 2016[20] | Prospective comparative study  USA | Adults with unresectable HCC and bilirubin ≤3.0 mg/dL | TheraSphere  (n=428) | TACE  (n=337) | Survival outcomes were stratified by Child-Pugh class and BCLC stage; patients with earlier stage disease appeared to have better survival outcomes with TACE than TheraSphere whilst patients with later stage disease appeared to have slightly better survival outcomes with TheraSphere than TACE. Progression-free survival, response rates and adverse event rates were not reported. | High |
| Maccauro, 2014[10] | Prospective comparative study  Italy | Adults with unresectable HCC (Child-Pugh A) | TheraSphere plus sorafenib  (n=15) | TheraSphere alone  (n=30) | There was no significant difference in overall survival or progression-free survival between treatment groups. Response rates were similar between treatment groups when assessed using mRECIST criteria, but favoured TheraSphere alone when assessed using EASL criteria (40% vs 10%). Adverse event rates were not reported. | High |
| Woodall, 2009[24] | Prospective comparative study  USA | Adults with unresectable HCC (including both patients with and patients without portal vein thrombosis) | TheraSphere in patients without PVT (n=20)  TheraSphere in patients with PVT (n=15) | Best supportive care/no treatment  (n=17) | Median overall survival was longer in TheraSphere patients without PVT (13.9 months) than TheraSphere patients with PVT (3.2 months) or patients on best supportive care (5.2 months). Progression-free survival and response rates were not reported. Adverse events were reported by more patients on TheraSphere with PVT (33%) than without PVT (25%). | High |
| Biederman, 2015[25] | Retrospective comparative study  USA | Adults with HCC with portal vein thrombosis | TheraSphere  (n=72) | SIR-Spheres  (n=25) | Median overall survival was longer with TheraSphere (15 months) than SIR-Spheres (4.1 months). Progression-free survival, response rates and adverse event rates were not reported separately for the different treatment groups. | High |
| Biederman, 2016[26] | Retrospective comparative study  USA | Adults with HCC with portal vein invasion | SIR-Spheres  (n=21) | TheraSphere  (n=69) | Median overall survival was statistically significantly longer with TheraSphere (9.5 months) than SIR-Spheres (3.7 months). Median time to progression was longer with TheraSphere (5.9 months) than SIR-Spheres (2.8 months). More patients had a complete or partial response with TheraSphere than SIR-Spheres, although only 57 TheraSphere patients and 15 SIR-Spheres patients were evaluable. The most commonly reported grade 3/4 adverse events were reported by more patients on SIR-Spheres than TheraSphere. | High |
| Van Der Gucht, 2017[28] | Retrospective comparative study  Switzerland | Adults with unresectable HCC | SIR-Spheres  (n=41) | TheraSphere  (n=36) | Overall survival and progression-free survival were similar between treatment groups. Response rates and adverse event rates were not reported. | High |
| Bhangoo, 2015[29] | Retrospective comparative study  USA | Adults with unresectable HCC | SIR-Spheres  (n=11) | TheraSphere  (n=6) | Overall survival was similar between treatment groups. Progression free survival was not reported. Response rates were not reported separately for the different treatment groups. The most commonly reported adverse events were reported by more patients on SIR-Spheres than TheraSphere. | Unclear |
| d’Abadie, 2018[27] | Retrospective comparative study  Belgium | Adults with HCC | SIR-Spheres  (n=33 procedures) | TheraSphere  (n=25 procedures) | Only Kaplan-Meier curves for different equivalent uniform doses were presented. | High |
| Radosa, 2019[30] | Non-comparative study  Germany | Adults with HCC | QuiremSpheres | N/A | Overall survival and progression-free survival were not reported. At 60 days 56% patients had a partial response, 33% had stable disease and 11% had progressive disease. At 6 months 11% had a complete response, 45% had a partial response, 33% had stable disease and 11% had progressive disease. There were 16 reportable adverse events in the 9 patients, but no grade 3/4 adverse events. | High |
