# Supplementary material for: Methods for selecting the best evidence to inform a NICE technology appraisal on selective internal radiation therapies for hepatocellular carcinoma
Source: Syst Rev. 2020 Aug 16;9:184. doi: 10.1186/s13643-020-01447-x (PMC7429468; doi:10.1186/s13643-020-01447-x)
Supplement: Supplementary file 2 — Additional file 2. Risk of bias assessment results for the 20 studies prioritised for data extraction. (DOCX) Additional file 2 is a table presenting the risk of bias assessment results for the 20 studies prioritised for data extraction. [file 13643_2020_1447_MOESM2_ESM.docx]

## Additional file 2: Risk of bias assessment results for the 20 studies prioritised for data extraction

Risk of bias assessment results for randomised controlled trials

| **Trial** | **Risk of bias arising from the randomisation process** | **Risk of bias due to deviations from the intended interventions** | **Missing outcome data (primary outcome)** | **Risk of bias in measurement of the outcome** | **Risk of bias in selection of the reported result** | **Overall judgement of risk of bias** |
| --- | --- | --- | --- | --- | --- | --- |
| Vilgrain, 2017[13]  SARAH | Low | Low | Low | Low | Low | Low |
| Chow, 2018[12]  SIRveNIB | Low | Low | Low | Low | Low | Low |
| Kolligs, 2015[14]  SIR-TACE | High | Low | High | High | Low | High |
| Pitton, 2015[18] | Some concerns | Low | Low | Low | Low | Some concerns |
| Ricke, 2015[15]  SORAMIC | Some concerns | High | Low | Low | Low | High |
| Salem, 2016[16]  PREMIERE | High | Some concerns | Low | Low | Low | High |
| Kulik, 2014[17] | Some concerns | Some concerns | Low | Some concerns | Low | Some concerns |

Risk of bias assessment results for prospective comparative studies

| **Study** | **Inclusion criteria clearly defined** | **Allocation to treatment groups adequately described/appropriate** | **Groups similar at baseline** | **Clearly described and consistently delivered intervention** | **Clearly described and consistently delivered comparator** | **Outcome assessors blinded** | **Missing outcome data balanced across groups** | **Free from suggestion of selective reporting** | **Overall judgement of risk of bias** |
| --- | --- | --- | --- | --- | --- | --- | --- | --- | --- |
| Kirchner, 2019[21] | No | No | No | Yes | No | No | Yes | Yes | High |
| El Fouly, 2015[19] | Yes | No | No | Yes | Yes | No | Yes | Yes | High |
| Salem, 2013[23] | Yes | No | No | Yes | Yes | No | Yes | Yes | High |
| Memon, 2013[22] | Yes | No | Yes | Yes | Yes | No | Yes | Unclear | High |
| Hickey, 2016[20] | Yes | No | No | Yes | Yes | No | Yes | Yes | High |
| Maccauro, 2014[10] | No | No | Unclear | No | No | Unclear | Unclear | Unclear | High |
| Woodall, 2009[24] | Yes | No | No | Yes | Yes | No | Yes | Yes | High |

Risk of bias assessment results for retrospective comparative studies

| **Study** | **Inclusion criteria clearly defined** | **Representative sample from relevant population** | **Groups similar at baseline** | **Clearly described and consistently delivered intervention** | **Clearly described and consistently delivered comparator** | **Outcome assessors blinded** | **Missing outcome data balanced across groups** | **Free from suggestion of selective reporting** | **Overall judgement of risk of bias** |
| --- | --- | --- | --- | --- | --- | --- | --- | --- | --- |
| Biederman, 2015[25] | No | Unclear | Unclear | No | No | Unclear | Unclear | Unclear | High |
| Biederman, 2016[26] | Yes | Yes | No | Yes | Yes | Unclear | Unclear | Unclear | High |
| Van Der Gucht, 2017[28] | Yes | Yes | No | Yes | Yes | Unclear | Yes | Yes | High |
| Bhangoo, 2015[29] | Yes | Yes | Unclear | Yes | Yes | Unclear | Unclear | Yes | Unclear |
| d’Abadie, 2018[27] | No | Unclear | No | No | No | Unclear | Unclear | Yes | High |

Risk of bias assessment results for non-comparative studies

| **Study** | **Inclusion criteria clearly defined** | **Representative sample from relevant population** | **Clearly described and consistently delivered intervention** | **Outcome measures pre-specified, reliable and consistently assessed** | **Outcome assessors blinded** | **Attrition low and accounted for in analysis** | **Incomplete outcome data minimal/dealt with in analysis** | **Overall judgement of risk of bias** |
| --- | --- | --- | --- | --- | --- | --- | --- | --- |
| Radosa, 2019[30] | Yes | Unclear | Yes | No | No | N/A (retrospective database of treated patients) | Yes | High |
